# Incongruity Between Knowledge and Preventive Practices on Hepatitis B Infection Among University Students in Northeastern, Tanzania

**DOI:** 10.24248/eahrj.v7i1.710

**Published:** 2023-07-12

**Authors:** Eward Erick, Kevin Rwegoshola, Pendo M. Ibrahim, Hadija Semvua, Jaffu Chilongola

**Affiliations:** a Department of Medical Biochemistry and Molecular Biology, Faculty of Medicine Kilimanjaro Christian Medical University College, Moshi Tanzania; b Kilimanjaro Clinical Research Institute, Moshi, Tanzania; c Department of Pharmacy, Kilimanjaro Christian Medical Centre, Moshi Tanzania

## Abstract

**Background:**

Young population is at high risk of acquiring sexually transmitted infections including hepatitis B virus, and thus the key target group for intervention. University students are reported to have inadequate knowledge concerning HBV. This study aimed to generate information on students' knowledge and attitudes surrounding HBV preventive practices.

**Methodology:**

A cross-sectional study was conducted in three Tanzanian universities in Moshi town of the northern Tanzanian region of Kilimanjaro. A total of 283 students were interviewed regarding their knowledge, attitudes, and practices regarding Hepatitis B Virus infection. Bloom's cut-off of 80% was used throughout to determine whether respondents had appropriate Knowledge, Attitudes, and Practices (KAP). Chi-squared test was used to measure independent associations between observed KAP levels with any demographic risk factors, with a *P* value of 0.05 as the cut-off for statistical significance.

**Results:**

There was a fairly good knowledge about HBV among students among the three universities such that; 22.3%, 33.9% and 43.8% of the students had good, moderate and poor knowledge about HBV, respectively. While 46.3% of the students showed neutral attitude towards HBV, 29.3% and 24.4% had positive and negative attitudes, respectively. Only 6.0% of the students had good practices for HBV whereas 21.6% and 72.4 showed moderate and poor practices respectively. With regards to good knowledge, associated demographic factors included: Being single (*P=.007)*; Having a master's degree *(P=.039*) and being a student at MWECAU (*P=.001*). Being single and being a student at MWECAU were also independently associated with positive attitude to HBV (*P=.007*) and (*.001*), respectively. No demographic factor was associated with HBV practices.

**Conclusions:**

The overall knowledge regarding HBV was fairly good among students from the three universities. Neutral attitude towards HBV demonstrated by the studied students may indicate stigma against HBV carriers. Notwithstanding the positive knowledge and the moderate attitude about HBV, there was an apparent poor practice towards HBV prevention especially vaccination and screening. Our findings, underscore the need to bridge the prominent gap between knowledge and practices among the high-risk youth in universities and schools by up scaling sensitization campaigns on preventive practices against HBV and other related viruses.

## BACKGROUND

Hepatitis B is a viral illness that targets the liver and causes both acute and chronic disease. Transmission occurs predominantly through contact with blood or body fluids of a person who is infected with the Hepatitis B virus (HBV). It also involves mother-to-child transmission (MTCT)) as well as sexual, sharing of sharp items and intravenous routes.^1–3^ People who are chronically infected are often unaware and asymptomatic, making it easy for them to spread the disease.^4, 5^ These patients are at a high risk of developing symptomatic liver disease, cirrhosis, and hepatocellular carcinoma, all of which can lead to mortality.^6–9^

Hepatitis B is a serious public health problem with a global prevalence that varies greatly between countries. Africa has the second highest burden of chronic hepatitis B.^10, 11^ Each year, almost 1.5 million people, predominantly young adults, become infected with the hepatitis B virus.^10^ Tanzania is considered as the highly endemic area for HBV with a prevalence of 7.17%.^12^ Sub-population studies in various sections of the country, however, revealed that the prevalence of HBV is 5.5-20%.^3^ There is also a significant exposure risk among adults in Tanzania, with Hepatitis B prevalence of 5% among the adult population.^13–15^

Most of university students are at high risk in terms of hepatitis B virus infection since they are more likely to engage in activities that put them at risk of hepatitis B infection,^6–9^ Unprotected sexual contact, sharing razors or toothbrushes, using contaminated needles when tattooing or body piercing, and being exposed to the blood of HBV carriers during clinical practice among medical students are all risky activities.^16, 17^ University students were previously reported to have inadequate knowledge and attitudes concerning HBV.^18–21^ In efforts to eradicate HBV in Tanzania by 2030, scaling up of public knowledge about the viral infection has been identified as one of the most effective.^22–24^ As a sexually transmitted infection, the young population from the start of puberty up to the age of marriage, remains the target group for prevention to maintain the initial success established in the management of HBV. Young people are also relatively easy to reach for preventive interventions via educational institutions where they spend many years of their early lives.

The practice of risky behaviours for contracting HBV among university students lags of knowledge and attitude about HBV awareness, knowledge and, consequently, prevention. Therefore, the purpose of this study was to understand how much the university community knew about HBV in relation to their preventive practices. This study was therefore designed to bridge this knowledge gap, using three Tanzanian universities in Moshi town of the northern Tanzanian region of Kilimanjaro.

## METHODOLOGY

### Study Setting

This study was conducted at three universities in Moshi, Tanzania namely Kilimanjaro Christian Medical University College (KCMUCo), Mwenge Catholic University (MWECAU), and Moshi Co-operative University (MOCU). This study location was chosen due to the reported increase in hepatitis B prevalence from 4.2% in 2016 to 5.7% in 2018.^25^ According to the National Bureau of Statistics (NBS) predictions for 2019, Moshi Municipal is home to 225,225 people.^26^ The main economic activities of locals are agriculture, business, and tourism.

### Study Design, Duration, and Population

This was a descriptive cross-sectional survey conducted between May and October 2021. The survey included at least 92 students from each of the three purposively selected universities. Except for those who declined to participate, all students who were on campus at the time of data collection and who willingly agreed to engage in the study were included in the sampling frame which was used to sample the desired number of participants in each university.

### Sample Size

Sample size was estimated by the “Epitools” online sample size (ss) calculator based on the formula ss= Z^2^(P) (1-P)/ε^2^, where ‘Z’ is the value (1.96 for 95% confidence level [CI]), ‘P’ represents prevalence (0.08),^27^ and ‘ε’ is the minimal tolerable error at 95% CI, expressed as a decimal (0.05). These estimations gave a minimum sample size of 114. However, in order to accommodate sampling error inherent to conveniently selected participants, the sample was increased to 283 participants. This increase of sample size was also meant to increase the statistical power. Out these 95, 96 and 92 participants were obtained from MWECAU, KCMUCo and MOCU, respectively.

### Sampling and Study Procedures

Purposive sampling of universities, followed by a random selection of participants was used to obtain study participants. The written consent form was presented to those students who were on campus when the data were being collected. The consent form was written in both English and Swahili and explained the study, its methods, and its advantages. Additionally, the research team made sure the participants were aware of the study's procedures. Before signing the consent, any queries from the participants were addressed. The consent form was signed by those who agreed to take part in the study and countersigned by the research team. A structured, self-administered questionnaire was then completed by the study participants who consented.

### Variables and their Categorisation

In this study, knowledge, attitude, and preventive measures for Hepatitis B infection were the dependent variables whereas socio-demographic characteristics such as age, gender, marital status, education, and occupation were the independent variables. Throughout the responses, categories for knowledge, attitudes and practices were based on the Bloom's cut-off criteria. For knowledge assessment, each correct answer received a one point, while each erroneous response received a zero score. Using Bloom's cut-off criterion, participants' overall knowledge, attitude and practice were rated as excellent if they were between 80 and 100%, moderate if they were between 60 and 79% and low if they were less than 60%.

### Quality Assurance

The reliability of the knowledge, attitude, and practice questionnaires was evaluated, and the Cronbach's alpha values were 0.71, 0.78, and 0.76, respectively, showing satisfactory internal consistency. Data was collected by four research scientists. Senior managers oversaw and regulated the entire data collection process. The supervisors examined the completed surveys for completeness and uniformity of responses. The questionnaires were changed as needed before data collection commenced.

### Data Management

Data were initially transferred to Microsoft Excel 365 for cleaning and coding. STATA version 15.1 was used to analyse the cleaned data. Frequencies and proportions were used to summarize categorical data. Bloom's cut-off of 80% was used throughout to determine whether respondents had appropriate knowledge (80%), attitudes and practices. To examine association between dependent variables (knowledge, attitude and practice) and independent variables (socio-demographic characteristics), a Chi-squared test was used.

### Ethical Consideration

Ethical clearance to carry out this study was obtained from the College Research Ethical Committee of Kilimanjaro Christian Medical University College (KCMUCo), ethical clearance #UG61/2022. Before taking part in the study, all participants were required to complete an informed consent form. Participants were able to refuse to participate in the study without providing any reason. Individual information was not disclosed to any third party. To preserve anonymity, all questionnaires did not include any personal identifiers.

## RESULTS

### Social demographic characteristic

There were 283 university students that participated in this study. Of these, 153 (54.1%) students were male with majority aged 20-34 years (98.6%). Slightly over half of the respondents (51.2%) were students of Diploma education level (Table 1)

**TABLE 1:** Socio-demographic Characteristics (N=283)

Variable	Frequency	Percentage
Sex		
Male	153	54.1
Female	130	45.9
Age in years		
< 19	2	0.7
20–34	279	98.6
> 35	2	0.7
Marital status		
Married	41	14.5
Cohabiting	44	15.6
Single / Divorced / Separated	198	69.9
Education level		
Diploma level	145	51.2
Degree level	134	47.4
Masters level	4	1.4
University		
MWECAU	95	33.6
KCMUCo	96	33.9
MOCU	92	32.5

**TABLE 2:** Knowledge of hepatitis B virus (N =283)

Variable	Frequency	Percentage
Carriers of hepatitis B can spread the virus of disease to others		
Yes	195	68.9
No	64	22.6
I don't know	24	8.5
Hepatitis B can be spread through casual contact such as holding hands		
Yes	171	60.4
No	79	27.9
I don't know	33	11.7
Hepatitis B can be spread through contact with open wounds/cuts		
Yes	201	71.0
No	51	18.0
I don't know	31	11.0
Hepatitis B can be transmitted by contaminated blood and blood products		
Yes	201	73.9
No	35	12.4
I don't know	39	13.7
Hepatitis B can be transmitted by un-sterilized syringes, needles, and surgical instruments		
Yes	176	62.2
No	68	24.0
I don't know	39	13.8
Hepatitis B Can be transmitted by unsafe sex		
Yes	198	70.0
No	62	21.9
I don't know	23	8.1
HBV has laboratory test		
Yes	236	84.3
No	23	8.2
I don't know	21	7.5
HBV vaccine prevent Hepatitis B		
Yes	233	83.2
No	22	7.9
I don't know	25	8.9
Hepatitis B virus causes liver cancer		
Yes	179	63.2
No	52	18.4
I don't know	52	18.4
Higher risk of living in the same house with someone infected with Hepatitis B		
Yes	178	62.9
No	73	25.8
I don't know	32	11.3
Suppose you are living in the same house with someone with Hepatitis B, what would you do to ensure you don't get infected		
Isolate infected person	32	11.3
Get vaccinated	228	80.6
Nothing	23	8.1

### Source of Information

**FIGURE 1:**
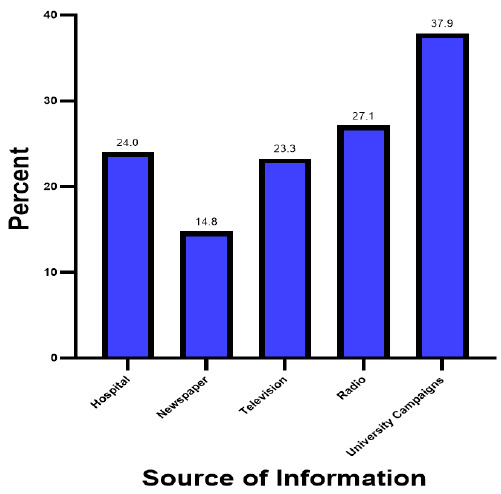
Source of information on Hepatitis B among University students in Kilimanjaro (N=274)

Almost all study participants 274 (96.8%) ever heard of Hepatitis B, of which 37.9% of these students had heard of Hepatitis B at their respective universities. Newspaper was the weakest source of information on HBV infection, informing only 14.8% of the participants as presented in Figure 1.

### Knowledge of Hepatitis B

**FIGURE 2:**
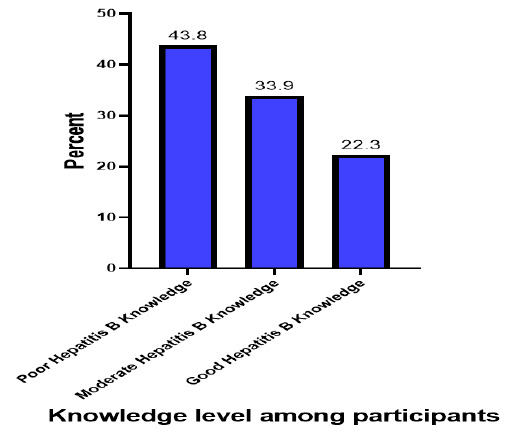
Knowledge Level on Hepatitis B among University students in Kilimanjaro (N=274)

About one-fifth (22.3%) of the participants had good knowledge on Hepatitis B whereas 43.8% of the participants had poor knowledge on Hepatitis B (Figure 2)

Students' knowledge on HBV varied with marital status, level of education and university type (Table 3).

**TABLE 3:** Knowledge on HBV by Social-demographic Characteristics (N= 283)

Variable	Total n (%)	Poor knowledgen (%)	Moderate knowledgen (%)	Good knowledgen (%)	χ2	P value
Sex						
Male	153 (54.1)	64 (51.6)	57 (59.4)	32 (50.8)	1.66	.436
Female	130 (45.9)	60 (48.4)	39 (40.6)	31 (49.2)		
Age < 19	2 (0.7)	1 (50.0)	0 (0.0)	1 (50.0)	2.814	.589*
20–34	279 (98.6)	122 (43.7)	96 (34.4)	61 (21.9)		
> 35	2 (0.7)	1 (50.0)	0 (0.0)	1 (50.0)		
Marital status						
Married	41 (14.5)	26 (63.4)	9 (22.0)	6 (14.6)	14.033	.007
Cohabiting	44 (15.6)	22 (50.0)	18 (40.9)	4 (9.1)		
Single / Divorced	198 (69.9)	76 (38.4)	69 (34.9)	53 (26.7)		
Education level						
Diploma level	145 (51.2)	60 (41.4)	47 (32.4)	38 (26.2)	10.630	.039*
Degree level	134 (47.35)	63 (47.0)	49 (36.6)	22 (16.4)		
Masters level	4 (1.4)	1 (25.0)	0 (0.0)	3 (75.0)		
University						
MWECAU	95 (33.6)	32 (33.7)	30 (31.6)	33 (34.7)	25.138	<.001
KCMUCo	96 (33.9)	41 (42.7)	30 (31.3)	25 (26.0)		
MOCU	92 (35.5)	51 (55.4)	36 (39.1)	5 (5.4)		

* P value by Fisher's exact estimation

### Attitude toward Hepatitis B

**FIGURE 3:**
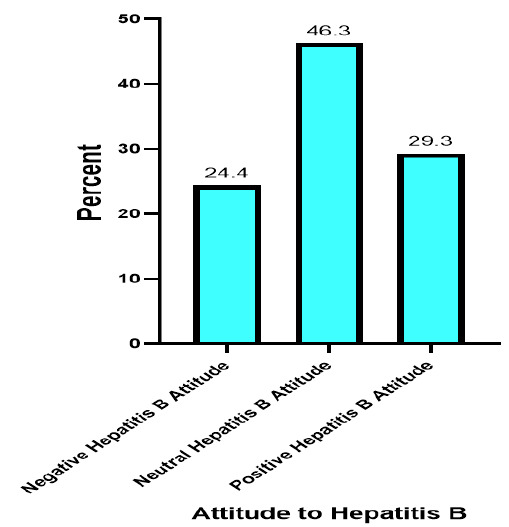
Attitudes toward Hepatitis B among University Students in Kilimanjaro, Tanzania (N=283)

A high percentage of students showed a neutral attitude toward Hepatitis B (46.3%) with 29.3% of the students showing a positive attitude toward Hepatitis B (Figure 3).

Table 4 summarises items used to assess the attitude toward Hepatitis B. Marital status and “university” differed significantly with knowledge of hepatitis B at *P*-value of < .05 as shown in Table 5.

**TABLE 4:** Attitude on Hepatitis B virus (N=283)

Variable	Yes n (%)	No n (%)	I don't know n (%)
Would you like to be given more information on Hepatitis B?	240 (84.8)	33 (11.7)	10 (3.5)
Do you think Hepatitis B is a curable disease?	187 (66.1)	73 (25.8)	23 (8.1)
Do you think Hepatitis B is a danger to your health?	242 (85.5)	29 (10.3)	12 (4.2)
Do you believe Hepatitis B infection is a serious health problem?	206 (72.8)	34 (12.0)	43 (15.2)
Do you think taking the Hepatitis B vaccine is safe?	215 (76.0)	31 (10.9)	37 (13.1)
Are you at risk of getting Hepatitis B?	146 (51.6)	94 (33.2)	43 (15.2)
Do you believe in the Hepatitis B vaccine?	209 (73.8)	39 (13.8)	35 (12.4)
Should all patients be tested for Hepatitis B before they receive health care?	199 (70.3)	63 (22.3)	21 (7.4)
Following Hepatitis B health guidelines will protect me from being infected.	210 (74.2)	56 (19.8)	17 (6.0)

**TABLE 5:** University Students' Attitude Toward HBV by Social-demographic characteristics (N= 283)

Variable	Total n (%)	Negative attitude n (%)	Neutral attitude n (%)	Positive attitude n (%)	χ^2^	P value
Sex						
Male	153 (54.1)	34 (49.3)	78 (59.5)	41 (49.4)	2.948	.229
Female	130 (45.9)	35 (50.7)	53 (40.8)	42 (50.6)		
Age						
< 19	2 (0.7)	1 (50.0)	1 (50.0)	0 (0.0)	1.916	.926*
20–34	279 (98.6)	68 (24.4)	129 (46.2)	82 (29.4)		
> 35	2 (0.7)	0 (0.0)	1 (50.0)	1 (50.0)		
Marital status						
Married	41 (14.5)	11 (26.8)	25 (61.0)	5 (12.2)	22.530	<.001
Cohabiting	44 (15.6)	20 (45.5)	17 (36.6)	7 (15.9)		
Single / Divorced	198 (69.9)	38 (19.2)	89 (44.9)	71 (35.9)		
Education level						
Diploma level	145 (51.2)	36 (24.8)	65 (44.8)	44 (30.4)	4.765	.429*
Degree level	134 (47.35)	33 (24.6)	65 (48.5)	38 (26.9)		
Masters level	4 (1.4)	0 (0.0)	1 (25.0)	3 (75.0)		
University						
MWECAU	95 (33.6)	14 (14.7)	42 (44.2)	39 (41.1)	29.425	<.001
KCMUCo	96 (33.9)	19 (19.8)	43 (44.8)	35 (35.4)		
MOCU	92 (35.5)	69 (24.4)	46 (50.0)	10 (10.9)		

* P value by Fisher's exact estimation

### Prevention Practice against Hepatitis B

**FIGURE 4:**
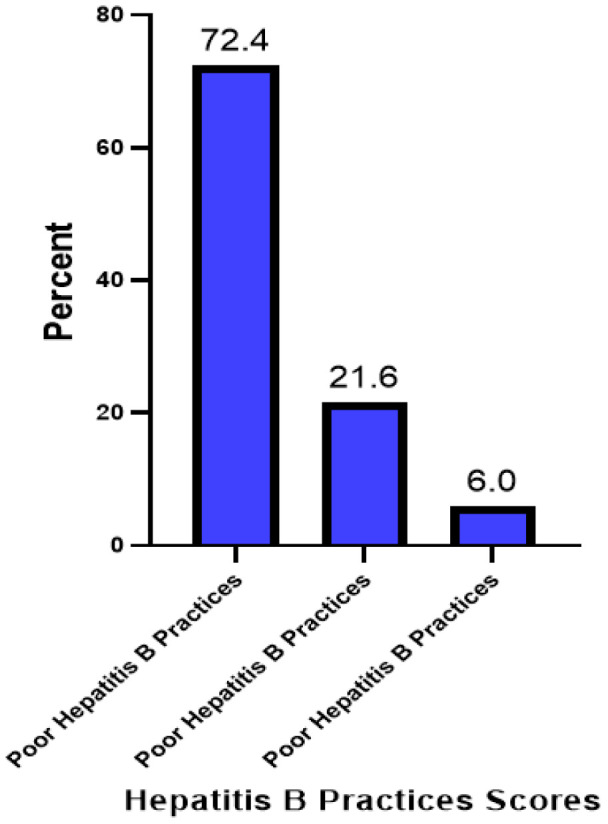
Overall Hepatitis B Prevention Practice among University Students in Kilimanjaro (N=283)

More than half of the participants had poor prevention practice against Hepatitis B (72.4%) Only 6% the students had good prevention practice against Hepatitis B (Figure 4). Items that were used to assess prevention practice against Hepatitis B are summarized in Table 6

**TABLE 6:** Preventive practices against Hepatitis B virus (N=283)

Variable	Frequency	Percentage
Ever done the screening for		
Hepatitis B		
Yes	120	42.6
No	156	55.3
I don't know	6	2.1
Ever Vaccinated against Hepatitis B		
Yes	95	35.6
No	181	63.9
I don't know	7	2.5
Willingness to be vaccinated (n=188)		
Yes	122	64.9
No	57	30.3
I don't know	9	4.8
Number of doses (n=95)		
One dose	32	33.7
Two doses	31	32.6
Three doses	32	33.7
Ever done injections with drug users		
Yes	2	0.7
No	281	99.3

Table 5 shows the proportional difference between social-demographic characteristics and prevention practices against Hepatitis B. None of the variables differed significantly with the prevention practices against Hepatitis B.

## DISCUSSION

HBV infection remains a serious threat to the public in our country although vaccine is available against it. According to the findings of this survey, most respondents had a positive attitude and knowledge about hepatitis B virus infection. They did, however, have a poor level of preventive practice for Hepatitis B virus infection. According to the findings of this study, only 61.1% of survey participants were knowledgeable of HBV, its mechanism of transmission, and prevention. The knowledge levels in this study are much lower than in previous studies conducted in Iran (90%), Vietnam (79.3%), and western Ethiopia (86.2 %).^28–30^ However, it was higher than reports from Ghana (63.9.5%) and Malaysia (50.3%).^31, 32^ The inclusion of both medical and non-medical students in our current study, as well as differences in the knowledge evaluation questions, could explain the discrepancy. In the current study, despite having a moderate awareness of the method of transmission, nearly one-tenth of the participants had no idea of how to live with an infected individual in the same home. This demonstrates the need of closing this gap by raising public understanding about how to avoid and control the spread of HBV.

This study had revealed that most of interviewed students had an appropriate knowledge that vaccination can effectively prevent hepatitis B. Despite this awareness, only 35.6% of respondents had confirmed receiving a dose of the HBV vaccine. Whereas a positive attitude towards HBV was recorded among 59.7% of the participants, various levels of attitudes have been reported elsewhere.^28–33^ Vaccines are known to be cost-effective in reducing the burden of several vaccine-preventable diseases, including HBV among high-risk and high-prevalence populations. The low vaccination rate reported in the current study is an alarm for increased efforts in sensitization of the highest risk youth in universities on the importance of vaccination against HBV. Moreover, most of the student participants were aware that HBV is serious disease with significant consequences and are more interested in learning more about it.

Health education through symposia and awareness campaigns has been shown to be an effective way to improve hepatitis B knowledge and attitude among university students.^32, 34, 35^ Our study is in concordance with other studies that emphasize unawareness, ignorance and inaccessibility of these vaccines in rural or peri-urban areas as barriers to HBV control.^32, 34, 35^ Despite the differences in study populations, our findings suggest that university students are receptive to hepatitis B health education. Although we observed a generally good attitude and level of awareness about HBV, our study participants' overall level of preventative practices was poor. In this example, less than half of the participants had ever been screened for HBV, typical evidence of poor practices. This is an obvious and worrisome gap between knowledge and practice regarding HBV.

Almost two-thirds (62.9%) of them would not want to live in the same house as HBV carriers. This discriminating rate is higher than predicted, which may be due to a lack of understanding of HBV transmission. This stigma can be eliminated by acquisition of appropriate knowledge about HBV and adopting a good attitude toward HBV infection. This urges universities and relevant government bodies such as the ministries of health and education to deliver positive guidance as soon as possible, to boost efforts to undertake peer education, and to promote relevant understanding the transmission pathways of HBV and other viruses to suppress the counterproductive stigma among university students.

Regarding hepatitis B practices among students, we report a poor level of preventive practices among students whereby 72.4%, 21.6% and 6% of the students had poor, moderate and good HBV practices respectively. This was a significant gap between knowledge and practices to HBV prevention. In terms of vaccination, 35.6% of research participants were immunized. However, out of the vaccinated students, just 33.7% (or only 11.3% of all students) received all three required dosages. Moreover, 42.6% of the participating students had ever screened for HBV.

## CONCLUSION AND RECOMENDATIONS

In summary, our data show that the overall knowledge regarding HBV, based on its mode of transmission and prevention was good among the three universities involved in this study. The overall attitudes towards HBV were mainly neutral but characterized with potential stigma against HBV carriers. Despite the positive knowledge and the moderate attitude about HBV, there was extremely poor practice towards HBV prevention especially vaccination and screening. Based on the apparent disparity between observed good knowledge regarding hepatitis B and practices, we recommend the organization of regular university campaigns that disseminate information on hepatitis B and related diseases as a strong means to create awareness among university students. Such campaigns will have far reaching impacts in the community outside university premises.

### Study Limitations

Although our study might have been minimally limited by recall bias due to the tool used to collect data, our findings, underscores the need to bridge the conspicuous gap between knowledge and practices among the high-risk youth in universities and schools by escalating sensitization campaigns on preventive practices against HBV and other related viruses.

## References

[1] Yuen MF, Balabanska R, Cottreel E et al. TLR7 agonist RO7020531 versus placebo in healthy volunteers and patients with chronic hepatitis B virus infection: a randomised, observer-blind, placebo-controlled, phase 1 trial. The Lancet Infectious Diseases 2023;23(4):496–507. 36509100 10.1016/S1473-3099(22)00727-7

[2] Iannacone M, Guidotti LG. Immunobiology and pathogenesis of hepatitis B virus infection. Nature Reviews Immunology 2022;22(1):19–32. 10.1038/s41577-021-00549-4 34002067

[3] Liu T, Song C, Zhang Y et al. Hepatitis B virus infection and the risk of gastrointestinal cancers among Chinese population: A prospective cohort study. International Journal of Cancer 2022;150(6):1018–28. 34855203 10.1002/ijc.33891 PMC9300134

[4] König A, Yang J, Jo E et al. Efficient long-term amplification of hepatitis B virus isolates after infection of slow proliferating HepG2-NTCP cells. Journal of Hepatology 2019;71(2):289–300. 31077792 10.1016/j.jhep.2019.04.010

[5] Conners EE, Panagiotakopoulos L, Hofmeister MG et al. Screening and testing for hepatitis B virus infection: CDC recommendations-United States, 2023. MMWR Recommendations and Reports 2023;72(1):1. 10.15585/mmwr.rr7201a1 PMC9997714 36893044

[6] Lim JK, Nguyen MH, Kim WR, Gish R, Perumalswami P, Jacobson IM. Prevalence of chronic hepatitis B virus infection in the United States. Official journal of the American College of Gastroenterology| ACG 2020;115(9):1429–38. 10.14309/ajg.0000000000000651 32483003

[7] Tan M, Bhadoria AS, Cui F et al. Estimating the proportion of people with chronic hepatitis B virus infection eligible for hepatitis B antiviral treatment worldwide: a systematic review and meta-analysis. The Lancet Gastroenterology & Hepatology 2021;6(2):106–19. 33197397 10.1016/S2468-1253(20)30307-1 PMC7801814

[8] Luo J, Liang X, Xin J et al. Predicting the Onset of Hepatitis B Virus-Related Acute-on-Chronic Liver Failure. Clinical Gastroenterology and Hepatology 2023;21(3):681–93. 35337983 10.1016/j.cgh.2022.03.016

[9] Alberts CJ, Clifford GM, Georges D et al. Worldwide prevalence of hepatitis B virus and hepatitis C virus among patients with cirrhosis at country, region, and global levels: a systematic review. The Lancet Gastroenterology & Hepatology 2022. 10.1016/S2468-1253(22)00050-4 PMC9259503 35576953

[10] Stockdale AJ, Kreuels B, Henrion MY et al. The global prevalence of hepatitis D virus infection: systematic review and meta-analysis. Journal of Hepatology 2020;73(3):523–32. 32335166 10.1016/j.jhep.2020.04.008 PMC7438974

[11] Froeschl G, Hoelscher M, Maganga LH et al. Hepatitis B, C and D virus prevalence in children and adults in Mbeya 10.11604/pamj.2021.39.174.26553 PMC8449578 34584600

[12] Region, Tanzania: results from a cohort study 2002-2009. Pan African Medical Journal 2021;39(1). 10.11604/pamj.2021.39.174.26553 PMC8449578 34584600

[13] Amponsah-Dacosta E. Hepatitis B virus infection and hepatocellular carcinoma in sub-Saharan Africa: Implications for elimination of viral hepatitis by 2030? World journal of gastroenterology 2021;27(36):6025. 34629817 10.3748/wjg.v27.i36.6025 PMC8476331

[14] Ansari A, Vincent JPt, Moorhouse L, Shimakawa Y, Nayagam S. Risk of early horizontal transmission of hepatitis B virus in children of uninfected mothers in sub-Saharan Africa: a systematic review and meta-analysis. The Lancet Global Health 2023;11(5):e715-e728. 37061310 10.1016/S2214-109X(23)00131-6

[15] Bernard Surial, Dominik Wyser, Charles Béguelin, Adrià Ramírez-Mena, Andri Rauch, Gilles Wandeler. Prevalence of liver cirrhosis in individuals with hepatitis B virus infection in subGÇÉSaharan Africa: Systematic review and metaGÇÉanalysis. Liver international 2021;41(4):710–9. 33220137 10.1111/liv.14744 PMC8048614

[16] Alves RF, Precioso JAG, Becoña EI. Sexual Knowledge and Attitudes and Sexual Risk Behaviors Among College Students. Egitania Sciencia 2022;83-102.

[17] Amare T, Yeneabat T, Amare Y. A systematic review and meta-analysis of epidemiology of risky sexual behaviors in college and university students in Ethiopia, 2018. Journal of environmental and public health 2019;2019. 10.1155/2019/4852130 PMC6446110 31015844

[18] Davies EL, Fielding S, Noble G, Okpo E. “It's just in that sea of things that I never cared about”: perception of hepatitis B amongst university students in Aberdeen, North-East Scotland. BMC public health. 2019;19(1):1–9. 30898127 10.1186/s12889-019-6654-z PMC6429810

[19] Shalal AAA-H. Assessment Of Nurses' - Knowledge Toward Hepatitis C Virus. Journal of Positive School Psychology 2022;6(9):4792–6.

[20] Gamal GA, Mohammed HE, Mohammed AH, Eltomy EM. Assessment of Knowledge & Practice regarding Hepatitis B/C infection risk and means of prevention among University Students.

[21] Abdi AM, Salleh MN. Knowledge, Attitude and Practice toward Prevention of Hepatitis B Virus Infection among Somalian Immigrant in the State of Selangor, Malaysia, and their HBV Infection Status. Journal of Bioscience and Applied Research 2019;5(2):198–211.

[22] Kazmi SK, Khan FMA, Natoli V et al. Viral hepatitis amidst COVID-19 in Africa: Implications and recommendations. Journal of Medical Virology 2022;94(1):7–10. 34506635 10.1002/jmv.27330 PMC8661579

[23] Ismail Z, Aborode AT, Oyeyemi AA et al. Impact of COVID-19 pandemic on viral hepatitis in Africa: Challenges and way forward. The International Journal of Health Planning and Management 2022;37(1):547–52. 34462959 10.1002/hpm.3317 PMC8653283

[24] MHINA J. Prevalence of hepatitis b vaccine and factors associated with vaccine uptake among healthcare workers in Mtwara municipality health centers, Tanzania. 2022.

[25] Shao ER, Mboya IB, Gunda DW et al. Seroprevalence of hepatitis B virus infection and associated factors among healthcare workers in northern Tanzania. BMC infectious diseases 2018;18:1–10. 30241503 10.1186/s12879-018-3376-2 PMC6151054

[26] Rugeiyamu R. The Tanzania Housing and Population Census 2022: A Panacea for Local Service Delivery and Development Drawbacks. Local Administration Journal 2022;15(1):1–13.

[27] Shedura VJ, Mchau GJ, Kamori D. High seroprevalence and associated risk factors for hepatitis B virus infection infection among pregnant women living with HIV in Mtwara region, Tanzania. Bulletin of the National Research Centre 2023;47(1):43.

[28] Pham TTH, Nguyen TTL, So S et al. Knowledge and Attitude Related to Hepatitis C among Medical Students in the Oral Direct Acting Antiviral Agents Era in Vietnam. International Journal of Environmental Research and Public Health 2022;19(19):12298. 36231600 10.3390/ijerph191912298 PMC9565151

[29] Aynalem A, Deribe B, Ayalew M et al. Practice towards Hepatitis B Virus Infection Prevention and Its Associated Factors among Undergraduate Students at Hawassa University College of Medicine and Health Sciences, Hawassa, Sidama, Ethiopia, 2021: Cross-Sectional Study. International Journal of Hepatology 2022;2022. 10.1155/2022/2673740 PMC9391155 35991003

[30] Hajare ST, Jemal SS, Alemu BD, Aseffa NM, Genet MM, Chauhan NM. Structural Equation Modeling for Magnitude, Association and Predictors of Knowledge, Attitude and Practice towards Hepatitis B among Tepi Community, Ethiopia. 2022.

[31] Otchere G, Dwomoh E, Kumah E et al. Knowledge, attitude and practice towards hepatitis B infection among high school students in Asante Mampong, Ghana. International Journal of Risk & Safety in Medicine 2022;33(3):269–79. 34719436 10.3233/JRS-200077

[32] Senoo-Dogbey VE, Wuaku DA. Risk perception for Hepatitis B Virus (HBV) infection among health care workers in Accra, Ghana. Clinical Epidemiology and Global Health 2022;18:101189.

[33] Ward VL, Tennermann NW, Chuersanga G et al. Creating a health equity and inclusion office in an academic pediatric medical center: Priorities addressed and lessons learned. Pediatric Radiology 2022;52(9):1776–85. 35229182 10.1007/s00247-022-05283-0 PMC8885314

[34] James TG, McKee MM, Sullivan MK et al. Community-engaged needs assessment of Deaf American Sign Language users in Florida, 2018. Public Health Reports 2022;137(4):730–8. 34161191 10.1177/00333549211026782 PMC9257506

